# Early surgery for strangulated and non-strangulated small bowel obstruction has no advantages over delayed procedure, especially in elderly patients: results of a multicenter retrospective cohort study

**DOI:** 10.1590/0102-672020260000041e1970

**Published:** 2026-10-09

**Authors:** Alexander TYAGUNOV, Anton TYAGUNOV, Taras NECHAY, Kirill MELNIKOV-MAKARCHUK, Zaripat ALIEVA, Botan ABDULKARIM, Alexander SAZHIN

**Affiliations:** 1 Pirogov Medical University, Pirogov Russian National Research Medical University, Surgery - Moscow, Russia.

**Keywords:** Intestinal Obstruction, Laparoscopy, Time Factors, Obstrução Intestinal, Laparoscopia, Fatores de Tempo

## Abstract

**Background:**

Adhesive small bowel obstruction (SBO) can be managed surgically or non-operatively. However, there are still no well-established criteria for defining the optimal treatment strategy. Recent evidence suggests that early intervention within 24 hours of admission to hospital may benefit patients.

**Methods:**

This was a multicenter retrospective study of adhesive SBO investigating the outcomes of early surgery (<12 hours), delayed surgery, and nonoperative treatment in 143 patients. In addition, the impact of time to surgery was also assessed in cases of strangulated and non-strangulated SBO.

**Results:**

Sixty-six (46.2%) patients underwent surgery, which was initiated laparoscopically in 49 (74.2%) cases. Of them, 36 (54.5%) patients were operated on within 12 hours of admission, and another 30 (45.5%) after 12 hours. The remaining 77 (53.5%) patients were successfully treated nonoperatively. In-hospital mortality was 7%, and all deaths (n=10; mean age 75.4 years, standard deviation±13.1 years) occurred postoperatively. Time from admission to surgery was not associated with mortality from any type of obstruction (strangulated or non-strangulated). Mortality was higher among older patients (>75 years) with non-strangulated SBO (p=0.01).

**Conclusions:**

The surgery performed in the first 12 hours of admission, whether for strangulated or non-strangulated adhesive SBO, did not show advantages over delayed surgery. A less aggressive approach to the treatment of older or frail patients would be a wiser option.

## ARTICLE HIGHLIGHTS

• Very early surgery (<12 hours) for adhesive small bowel obstruction does not improve mortality or clinical outcomes compared to delayed surgery.

• Time from admission to surgery was not associated with mortality in either strangulated or non-strangulated obstruction.

• Mortality occurred exclusively in surgical patients and was significantly higher among elderly individuals (>75 years).

• A less aggressive, nonoperative-first approach may be safer for frail and elderly patients with adhesive small bowel obstruction.

## Summary

**CENTRAL MESSAGE:**

Abdominal adhesions are the most common cause of bowel obstruction and place a substantial burden on healthcare systems due to their association with hospitalizations, surgeries, a mortality rate of 4-7%, and a high risk of recurrence. There is still no consensus on the accurate diagnostic criteria for early surgery and the safety of nonoperative management. Although nonoperative management is recommended as a safe initial treatment option, many surgeons seek to speed up surgery due to the risk of strangulation. Very early surgery for adhesive small bowel obstruction (SBO) performed within 12 hours of admission did not benefit patients in this study. A less aggressive approach to the management of frail patients with adhesive SBO would be a wiser option than early surgery prompted by the overestimating of strangulation.

**PERSPECTIVES:**

There are still insufficient data to draw an overarching conclusion about optimal SBO management. Further randomized controlled trials and studies based on standardized SBO evaluation are needed.

## VISUAL ABSTRACT

**Figure:**
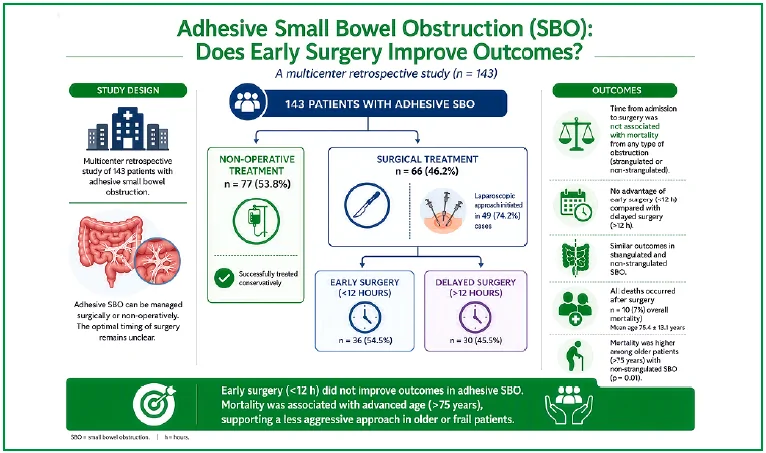
VISUAL ABSTRACT

## RESUMO

**Racional:**

A obstrução de intestino delgado (OID) por aderências pode ser manejada de forma cirúrgica ou não operatória. Contudo, ainda não existem critérios bem estabelecidos para definir a melhor estratégia terapêutica. Evidências recentes sugerem que a intervenção precoce, realizada até 24 horas após a admissão, poderia trazer benefícios aos pacientes.

**Métodos:**

Este estudo retrospectivo multicêntrico de OID por aderências investigou os desfechos de cirurgia precoce (<12 horas), cirurgia tardia e tratamento não operatório em 143 pacientes. Também foi avaliado o impacto do tempo até a cirurgia em casos de OID estrangulada e não estrangulada.

**Resultados:**

Sessenta e seis pacientes (46,2%) foram submetidos à cirurgia, iniciada por laparoscopia em 74,2% dos casos. Entre eles, 36 (54,5%) foram operados em menos de 12 horas da admissão e 30 (45,5%) após 12 horas. Os demais 77 pacientes (53,5%) tiveram resolução clínica sem cirurgia. A mortalidade intra-hospitalar foi de 7%, e todos os óbitos ocorreram no pós-operatório (n=10; idade média 75,4 anos, desvio padrão±13,1 anos). O tempo entre a admissão e a cirurgia não se associou à mortalidade para nenhum tipo de obstrução (estrangulada ou não estrangulada). A mortalidade foi maior em pacientes idosos (>75 anos) com OID não estrangulada (p=0,01).

**Conclusões:**

A cirurgia realizada nas primeiras 12 horas, seja para OID por aderências estrangulada ou não estrangulada, não demonstrou vantagem sobre a intervenção tardia. Em pacientes idosos ou frágeis, uma abordagem menos agressiva pode ser mais segura.

## INTRODUCTION

Abdominal adhesions are the most common cause of bowel obstruction and place a burden on healthcare systems due to their association with hospitalizations, surgeries, a 4-7% mortality rate, and a high risk of recurrence^7^ ^,^ ^12^ ^,^ ^14^. There has been a gradual decline in mortality from adhesive small bowel obstruction (SBO) in the past decades, associated with improved diagnostics and a more thoughtful choice of management strategy^14^, but there is still no consensus on the accurate diagnostic criteria for early surgery and the safety of nonoperative management (NOM)^22^.

Although NOM is recommended as a safe initial treatment option^5^, recent research suggests it does not have any obvious advantages over early surgery^7^ ^,^ ^8^ ^,^ ^22^. According to the literature, surgical interventions performed within 24 h after presentation have better short-term outcomes^21^ and are cheaper than initial NOM^2^. At the same time, the evidence suggesting that delayed surgery has a negative effect on patients is not compelling, even in the case of strangulated obstruction^3^. There are only scarce data on how time to surgery may affect the outcomes in patients who do not need emergency surgery. Therfore, it might be useful to focus on comparing the outcomes of early (<24 h) *versus* delayed surgery^8^ ^,^ ^10^. Theoretically, surgery performed even earlier may offer some additional benefits for patients with adhesive SBO.

The aim of this multicenter study was to compare the outcomes of early (within 12 h of admission) *versus* delayed (following a failed nonoperative treatment) surgery in patients with adhesive SBO.

## METHODS

This retrospective observational cohort study included 143 consecutive patients with adhesive SBO treated from May 2017 to December 2019 at four Moscow hospitals. Non-adhesive bowel obstruction and early postoperative adhesive bowel obstruction were excluded from the analysis (Figure 1). No other exclusion criteria were applied.

**Figure 1.**
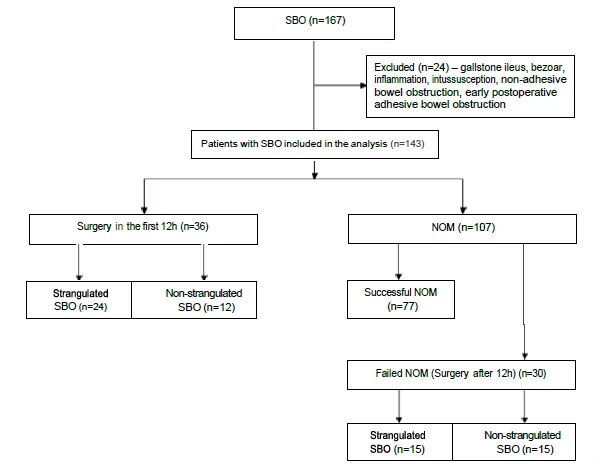
Flowchart showing patient selection and stratification by surgery timing.

SBO: small bowel obstruction; NOM: nonoperative management.

Surgery or NOM. The decision to attempt NOM or perform early surgery was guided by the clinical suspicion of strangulated SBO. Prior to the study, all the participating surgeons were informed about the risks and advantages of early surgery and NOM and the diagnostic value of laboratory and imaging tests (computed tomography) for suspected strangulated bowel obstruction. In addition, the surgeons were expected to follow the Russian clinical guidelines, which did not recommend continuing NOM beyond 12 h at the time of this study. However, several surgeons continued NOM for longer periods in the absence of obvious signs of strangulated obstruction.

Analysis of patients’ data. The patients’ data were saved to a database for further analysis. Retrospectively, early surgery was defined as a surgical intervention performed within 12 h of admission. Delayed surgery was defined as a surgical intervention performed later than 12 h of admission. The analysis revealed that both types of obstruction (strangulated and non-strangulated) occurred in both the early surgery group and the delayed surgery group (Figure 1). The impact of time to surgery on in-hospital mortality, the rate of complications and the length of hospital stay was evaluated in operated patients, including separately in the patients with strangulated and non-strangulated SBO.

After data analysis, a literature review of the relevant single-center observational studies was conducted. The results of the present analysis were compared with the findings of the most cited research studies with a similar design. Comparisons were made by age, disease severity, the rate of intestinal strangulation, the rate of bowel resection, the duration and effectiveness of NOM, and mortality among operated and non-operated patients.

This study was approved by the Ethics Committee of Pirogov Russian National Research Medical University (nº 258). The patients gave their informed consent to participate in the study and to have their personal data processed by the authors.

### Statistical analysis

The significance of differences between the mean values of quantitative data was measured using analysis of variance (ANOVA). The distribution of categorical variables was assessed with a χ^2^ test. Pairwise comparisons of categorical variables were made using Fisher’s exact test. Survival was estimated by the Kaplan-Meier method. The significance threshold was set at p<0.05.

The retrospective analysis revealed that surgery was performed on 66 (46.2%) patients; of them, 36 (54.5%) underwent surgery within 12 h of admission and another 30 (45.5%) after 12 h (failed NOM). Strangulated SBO was diagnosed in 39 patients; of them, 24 (61.5%) underwent surgery within 12 h of admission and 15 (38.5%) were operated on later. There were 12 patients with non-strangulated SBO who had early surgery because of clinical suspicion of strangulation. The remaining 15 patients with non-strangulated SBO underwent surgery after more than 12 h of unsuccessful NOM.

## RESULTS

A total of 68 male and 75 female patients participated in the study. Their mean age was 59.7 years (standard deviation±17.4 years). The majority of patients underwent conventional diagnostic laboratory tests for strangulation; computed tomography was performed in 78 (54.5%) patients and water-soluble contrast challenge was performed in 105 (69.9%).

The median time from admission to surgery was 12 h (interquartile range [IQR] 6-20). Fifty-two (78.8%) patients were operated on within 24 h of admission. The procedure was initiated laparoscopically in 49 (74.2%) patients; 13 (19.7%) required conversion to open surgery, whereas 17 (25.8%) underwent primary laparotomy. The mean operative time was 98.3±43.9 min. NOM was successful in 77 (53.8%) cases.

### Complications and mortality

Postoperative complications developed in 17 (25.8%) patients. Ten (7%) patients died. Their mean age was 75.4±13.1 years. All deaths (n=10) occurred after surgery performed within 24 h of admission. Other 14 (21.2%) patients who were operated at a later time point survived (p=0.192). No deaths occurred in the nonoperative treatment group. Although NOM had considerable advantages over both delayed and early procedures (Figure 2), the patients in the surgery group were older (p=0.013), and there were more patients with severe disease and higher Acute Physiology and Chronic Health Evaluation (APACHE) II (p<0.001) and American Society of Anesthesiology (ASA) scores than in the NOM group (p=0.024).

The cumulative probability of death (95% confidence interval [CI]) was estimated using the Kaplan-Meier method. The significance of differences between the groups is associated with the absence of deaths in the NOM group and increases to p=0.167 when the NOM group is excluded from the analysis.

**Figure 2.**
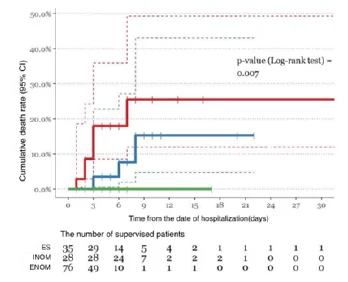
Cumulative probability of death associated with early surgery, delayed surgery and nonoperative management.

95%CI: 95% confidence interval; ES: early surgery; INOM: Initial nonoperative management; ENOM: exclusive nonoperative management.

### Comparison of early (<12 h) and delayed (>12 h) surgery

These groups were overall comparable, except that, patients in the early surgery group were younger (p=0.018) and the number of prior surgical interventions was higher (p=0.016). No differences were detected between these two groups in terms of mortality, strangulation, bowel resection, complications, and length of hospital stay (Table 1).

**Table 1.** Comparison of early and delayed surgery groups (n=66).

Parameter	Surgery in the first 12h n=36	Surgery after 12h n=30	p-value
Age, years	57.2±19.6	68.0±15.5	0.018
Sex, female/male	20/16	17/13	0.928
Prior surgeries, number	1.4±1.0	2.1±1.2	0.016
Median time from onset of symptoms to admission, hours	12 [1-120]	24 [2-264]	0.056
APACHE II score, points	11.3±5.6	13.0±4.7	0.176
APACHE II score >15 points	7 (20.0%)	8 (26.7%)	0.525
ASA class 1-2	28 (80.0%)	21 (70.0%)	0.351
ASA class 3-4	7 (20.0%)	9 (30.0%)	
Constant pain, n (%)	18 (52.9)	14 (48.3)	0.712
Constipation >24 h, n (%)	7 (25.0)	10 (52.6)	0.053
Presence of free intraperitoneal fluid, n (%)	22 (61.1)	15 (50.0)	0.294
Intestinal loop diameter, mm	35.6±4.9	33.3±7.3	0.233
White blood cells, ×10⁹/L	11.2±3.1	10.8±4.3	0.699
Lactate >2, mmol/L	8 (53.3%)	8 (38.1%)	0.364
Computed tomography, n (%)	20 (55.6)	16 (53.3)	0.857
Water-soluble contrast challenge, n (%)	6 (16.7)	18 (60.0)	<0.001
Time from admission to surgery, hours	6.6±3.6	30.1±22.6	<0.001
Initial laparoscopy, n (%)	21 (58.3)	28 (93.3)	0.472
Conversion to open surgery, n (%)	7 (33.3)	6 (21.4)	0.350
Intestinal strangulation, n (%)	24 (66.7)	15 (50.0)	0.170
Operative time, minutes	91.7±45.5	108.7±41.3	0.150
Bowel resection, n (%)	3 (8.3)	3 (10.0)	0.815
Postoperative complications, n (%)	9 (25.0)	8 (26.7)	0.877
Reoperation, n (%)	2 (5.6)	2 (6.7)	0.851
Length of hospital stay, days	6.2±5.7	8.0±4.2	0.163
Death, n (%)	7 (19.4)	3 (10.0)	0.327

±: standard deviation; APACHE: Acute Physiology and Chronic Health Evaluation: ASA: American Society of Anesthesiology.

### Early *versus* delayed surgery for strangulated small bowel obstruction

In these two groups (n=39; Table 2), the median time from the onset of symptoms to surgery was 29 h (IQR 14-56), and the median time from admission to surgery was 11 h (IQR 2-75). The groups differed only in the number of prior surgeries (p=0.039). Delayed surgery was not associated with worse outcomes (Figure 1), except for a longer hospital stay (p=0.039). The rate of bowel resection (p=0.617), mortality (p=0.940) and cumulative mortality by day 10 of hospital stay (13.0% [95%CI 12.0-14.1] *versus* 15.4% [95%CI 13.2-17.5]; p=0.860) were similar between the groups. Seven (17.9%) patients underwent surgery after 24 h in hospital; none of them died.

**Table 2.** Early *versus* delayed surgery for strangulated small bowel obstruction.

Parameter	Surgery within the first 12 h (n=24)	Surgery after 12 h (n=15)	p-value
Age, years	54.5±16.0	64.8±19.1	0.079
Sex, female/male	9/15	7/8	0.571
Prior surgeries, n (median)	1.1±1.0 (1.0)	1.9±1.4 (1.5)	0.039
Time from onset of symptoms to admission, hours (median)	8.0 (1-120)	20.0 (3-264)	0.074
APACHE II scores >15 points	2 (8.7%)	4 (26.7%)	0.138
ASA class 3 and 4	3 (13.0%)	4 (26.7%)	0.290
Constipation >24 hours	4 (21.1%)	6 (60.0%)	0.036
Presence of free intraperitoneal fluid, n (%)	12 (66.7)	9 (69.2)	0.880
Intestinal loop diameter >40 mm, n (%)	2 (15.4)	1 (12.5)	0.854
White blood cells, ×10⁹/L	11,0±3,2	10,8±4,6	0.847
Lactate >2, mmol/L	4 (40.0%)	5 (62.5%)	0.343
Computed tomography	14 (58.3%)	5 (33.3%)	0.129
Time from admission to surgery, hours	6.3±3.9	33.6±29.9	<0.001
Initial laparoscopy	20 (83.3%)	10 (66.7%)	0.229
Conversion to open surgery	4 (20.0%)	4 (40.0%)	0.243
Bowel resection, n (%)	2 (8.3)	2 (13.3)	0.617
Postoperative complications, n (%)	5 (20.8)	3 (20.0)	0.950
Reoperation, n (%)	1 (4.2)	1 (6.7)	0.731
Transfer to intensive care unit, n m(%)	9 (37.5)	9 (60.0)	0.170
Length of stay in ICU, hours	64.2±54.4	61.3±38.2	0.898
median	48.0	48.0	
Length of hospital stay, days	5.5±3.2	8.2±4.2	0.039
Death n (%)	3 (12.5)	2 (13.3)	0.940

APACHE: Acute Physiology and Chronic Health Evaluation; ASA: American Society of Anesthesiology; ICU: intensive care unit.

### Early *versus* delayed surgery for non-strangulated small bowel obstruction

Twenty-seven (26.0%) of the patients with strangulated SBO were operated within a mean of 18 [4; 48] h of admission; 74% were operated within 24 h. The groups (<12 h and >12 h) did not differ in their baseline characteristics except for higher lactate in the early surgery group (p=0.026). The outcomes did not correlate with surgery timing, although mortality tended to be higher among the patients who had early surgery (p=0.076) (Table 3). Postoperative mortality was especially high among the old patients: 5 out of 12 patients aged >75 years died. The rest 15 patients were younger; none of them died (p=0.01). The age of the non-survivors in the early surgery group was >80 years.

**Table 3.** Early *versus* delayed surgery for non-strangulated small bowel obstruction.

Parameter	Within the first 12 h (n=12)	After 12 h (n=15)	p-value
Age, years	62.7±25.2	71.1±10.5	0.248
Sex, female/male	5/7	9/6	0.343
Prior surgeries, n	2.0±0.9	2.3±1.1	0.497
Prior bowel obstruction episodes,	0.5±0.8	0.5±0.6	0.912
median	0 (0-2,0)	0 (0-2,0)	
Time from onset of symptoms to admission, hours (median)	22.0 (8-48)	24.0 (2-144)	0.372
APACHE II score >15 points	5 (41.7%)	4 (26.7%)	0.411
ASA class 3 and 4	4 (33.3%)	5 (33.3%)	1.000
Constant pain, n (%)	5 (50.0)	4 (26.7)	0.234
Constipation >24 hours, n (%)	3 (33.3)	4 (44.4)	0.629
Free intraperitoneal fluid on US scan, n (%)	8 (66.7)	6 (50.0)	0.117
Intestinal loop diameter on US scan >40 mm	0 (0.0%)	2 (20.0%)	0.156
White blood cells, ×10⁹/L	11.5±3.0	10.9±4.1	0.657
Lactate >2,mmol/L	4 (80.0%)	3 (23.1%)	0.026
Computed tomography, n (%)	6 (50.0)	11 (73.3)	0.212
Water-soluble contrast challenge, n (%)	4 (33.3)	9 (60.0)	0.168
Length of hospital stay, days (median)	5 (2-32)	7 (3-22)	0.917
Bowel resection, n	1	1	1.000
Death, n (%)	4 (33.3)	1 (6.7)	0.076

±: standard deviation; APACHE: Acute Physiology and Chronic Health Evaluation; ASA: American Society of Anesthesiology; US: ultrasound.

## DISCUSSION

Although the modern concept of SBO management proposed by World Society of Emergency Surgery (WSES)^5^ places a substantial focus on nonoperative treatment, the safety of the late surgical option may be an issue in case NOM is unsuccessful. There is evidence that early surgery performed within 24 h of admission is more beneficial for the patient^2^ ^,^ ^11^ ^,^ ^13^. In this study, we have attempted to identify the advantages of even earlier surgery for SBO (<12 hours after presentation) that have not been reported previously. A fair number of early surgeries are performed because of the clinical suspicion for strangulated bowel obstruction, which to this day remains a major diagnostic challenge.

The patients’ characteristics, approaches to treatment, and outcomes of early and delayed surgeries for SBO were compared using data from this and other observational studies identified (Table 4). Only some of them aimed to investigate the role of surgery timing and NOM duration and reported the rate of bowel resection, its cause, the rate of complications, and mortality. There was a significant variation between the analyzed studies in terms of duration and success of NOM, the type of surgery (laparoscopy or laparotomy), and some other parameters. Because of different designs, limited data on the outcomes, and different patient characteristics such as mean age (50 to 74 years), only partial comparison was possible. The number of early surgeries for SBO was especially high in the current study: 55% of the patients were operated within 12 h of admission. It appears that the strategy of early intervention was pursued in three more studies^2^ ^,^ ^6^ ^,^ ^15^, in which about 80% of patients were operated within 24 h of admission or the time to late operation was similar. In the current study, laparoscopy was performed in 54.5% of the patients, but there were only two out of nine analyzed studies where the procedure was laparoscopic^4^ ^,^ ^11^. Unlike the analyzed publications, there were no reports of anastomotic leaks or ostomies in the current study^1^ ^,^ ^18^ ^,^ ^20^.

**Table 4.** Results of small bowel obstruction treatment reported by different studies.

Study	n	ASA class 3, %	Surgery, %	Surgery in the first 24 h, %	Time o surgery, days	Strangulated obstruction, %	(%)* of bowel resection
Leung and Vu^13^ (USA)	872	n/a	57	80	1.69	n/a	15
Bauer et al.^1^ (USA)	460	n/a	78	30	n/a	13	28
Miller et al.^16^ (Canada)	675	25	30	n/a	n/a	6	20
Meier et al.^15^ (Switzerland)	221	n/a	61	88	n/a	n/a	32
Joseph et al.^11^ (USA)	85	n/a	100	n/a	n/a	n/a	n/a
Tabchouri et al.^20^ (France)	154	21	32	46	n/a	n/a	18
Fevang et al.^6^ (Norway)	109	n/a	44	12.8	1.1**	11	19
Sakari et al.^18^ (Sweden)	402	48	100	19	3.5	n/a	35
Darbyshire et al.^4^ (UK)	299	n/a	100	29	1.8	n/a	18
Current study (Russia) (IQR)	143	16	46	78	0.5 (0.25; 0.83)	26	9

*Percentage of operated patients; **only late operated group; ASA: American Society of Anesthesiology; n/a: not available; IQR: interquartile range.

### Surgery timing and mortality

Overall mortality (Table 5) was similar between the studies, except for the current study, although time to surgery varied. Postoperative mortality differed significantly between the studies and was generally higher than mortality associated with NOM. The majority of non-survivors were elderly patients (mean age: 73-82 years)^17^. Three studies, including the current study, that examined the impact of surgery timing on the outcome reported that it did not affect mortality^1^ ^,^ ^11^. By contrast, the study by Tabchouri et al.^20^ demonstrated the advantage of early surgery. However, the low overall mortality (3.9%) reported by Tabchouri et al.^20^ seems to be the result of the successful prolonged NOM in frail elderly patients (mean age: 76 years) rather than the benefit of early surgery. The outcome of very early surgery in the current study was not better than that of later surgery.

**Table 5.** Comparison of age and mortality among published studies on small bowel obstruction.

Study	Age, years				Mortality, %		
	Mean	Surgery	NOM	Deaths	NOM	PO	Total
Leung and Vu^13^ (USA)	50	47	50	n/a	n/a	n/a	n/a
Bauer et al.^1^ (USA)	60	57-60	60	n/a	1.0	2.8	2.4
Miller et al.^16^ (Canada)	60	60	63	n/a	n/a	3.0	n/a
Meier et al.^15^ (Switzerland)	67	68	65	77	0	6.6	4.1
Joseph et al.^11^ (USA)	n/a	n/a	n/a	n/a	n/a	1.1	n/a
Tabchouri et al.^20^ (France)	74	69-70	76	n/a	0	12.0	3.9
Fevang et al.^6^ (Norway)	58	n/a	n/a	n/a	1	6.3	3.7
Sakari et al.^18^ (Sweden)	70	70	n/a	82	n/a	5.2	n/a
Darbyshire et al.^4^ (UK)	70	70	n/a	73	n/a	3.0	n/a
Current study (Russia)	60	67	58	75	0	15.2	7.0

PO: postoperative; Total: in the pooled group of patients; NOM: nonoperative management; n/a: not available.

### Bowel resection rate

The rate of bowel resection varied from 1.7- to 4-fold between the studies (resection was performed on 9 to 35% of the operated patients). The causes of bowel resection were not provided, except for the study by Fevang et al.^6^. It is not safe to conclude that surgery timing affected the rate of bowel resection in the analyzed studies. Although the mean time to surgery was the shortest and the rate of bowel resection was the lowest in the current study, Meier et al.^15^, who performed early surgery on 88% of their patients, reported a rate of bowel resection that was three times higher than that reported in the current study and similar to the rate of resection in the studies with longer NOM^18^. The increased rate of bowel resection associated with delayed surgery was detected in one out of four studies that included this parameter in the analysis^13^. Based on the present analysis, the high rate of bowel resection did not correlate directly with a rise in mortality (Table 4).

### Complications

A significant rise in the rate of complications after surgery performed at >48 h was reported by one study^11^. According to Sakari et al.^18^, the rate of reoperation by laparotomy was higher for surgeries performed after 48 h. Surgeries performed after 96 h were more often complicated by wound dehiscence or led to a reoperation by laparotomy, but they were not associated with an increased rate of complications^18^. The increased rate of complications after early surgery was reported by Fevang et al.^6^.

### Strangulated bowel obstruction

Strangulated SBO was reported by four studies and occurred in 6 to 27% of patients; this broad variation might be explained by the use of different criteria for strangulation. In the early study^19^, strangulation occurred in 23% of the patients with SBO.

The rate of irreversible bowel ischemia differed significantly between the studies. In a study by Fevang et al.^6^, 75% of patients with strangulated SBO had nonviable bowel, in comparison with 10% in the current study. In another recent study, viable bowel, reversible bowel ischemia, and irreversible bowel ischemia occurred in 18.9, 58.1 and 23% of patients with closed loop obstruction, respectively^23^. Such differences can be explained by insufficient standardization of the anatomical definitions of SBO. The recently proposed American Association for the Surgery of Trauma (AAST) grading system for Adhesive Small Bowel Obstruction (ASBO) may be the solution and could be used to accurately compare the outcomes between different patient cohorts^9^.

The primary challenge of SBO is its early diagnosis. Although strangulated SBO is a direct indication for emergency surgery, the accuracy of its early diagnosis is low. In the study of closed-loop obstruction^23^, computed tomography was suspicious for ischemic bowel in 16.9% of the patients but inconclusive and unsuspicious for ischemia in 52.7 and 30.4% of the patients, respectively. Recent research has transformed the understanding of the negative impact of delaying surgery on the outcome of strangulated SBO. Bauer et al.^1^ reported that 40 (66%) of their patients with SBO were subjected to surgery later than 24 h after admission but there was no rise in the rate of bowel resection or mortality. In the current study, the surgeons attempted to make an early diagnosis of strangulated bowel obstruction following the World Society of Emergency Surgery (WSES) guidelines. As a result, two-thirds of the patients with strangulated SBO were operated on within 12 h of admission and 82.1% of the patients underwent surgery within 24 h of admission. In other words, the early detection of strangulated SBO was either more frequent or comparable to that reported by other studies. However, the only advantage of early surgery over delayed (after 12 h) surgery was a slightly shorter hospital stay (p=0.039).

### Surgery for non-strangulated SBO

Much more is written in research studies and guidelines about the operative management of strangulated than about non-strangulated bowel obstruction. Non-strangulated SBO deserves more attention; according to the present estimates, it occurred in 41 to 83% of the patients undergoing surgery. The only study that provided some information about the outcomes of non-strangulated SBO and thus allowed calculation of the mortality rate (8.3%) was the Fevang study. In the current study, mortality was 18.5%; the majority of deaths occurred in the early surgery group.

Based on four studies, including the current study, the proportion of patients operated for non-strangulated SBO was calculated: it varied between 26 and 75% (Table 4)^1^ ^,^ ^6^ ^,^ ^8^. In the Bauer et al.^1^ study, surgery was performed on 75% of the patients with non-strangulated SBO. The authors suggested that NOM was unsuccessful because their study included patients with complete SBO only. In three other studies, less than 40% of the patients with non-strangulated SBO were treated surgically, which was only slightly different from the current study. In the current study, though, 74% of the patients with non-strangulated SBO underwent surgery within 24 h of admission (44% were operated within 12 h), whereas the proportion of such patients in the Bauer et al.^1^ and Fevang e al.^6^ studies was 17 and 22%, respectively. Although the patients with non-strangulated SBO were often operated early because of clinical suspicion for strangulated SBO, surgery timing varied significantly between the studies. Nevertheless, this difference cannot explain the high mortality among the patients with non-strangulated SBO because similar percentage of early surgeries for this condition was reported by a few other studies where mortality was lower^15^. In contrast to other studies^20^, the surgeons participating in the current study chose surgery over NOM for the elderly and frail patients more often, which led to very high mortality in the early surgery group.

Thus, shorter time from presentation to surgery did not benefit the patients in the current study. Due to the substantial heterogeneity of the data provided by the retrospective studies included in the analysis, it is impossible to determine the optimal treatment strategy, surgery timing, NOM duration, etc. Although functionally intact patients may benefit from early surgery, NOM is probably the preferred treatment option for the elderly. It would be more rational to avoid overestimating the probability of strangulated SBO in elderly patients and subjecting them to early surgery.

### Study limitations

This retrospective study was conducted in a small group of patients; therefore, its conclusions may have limited practical application. The authors did not have access to the information about recurrences or readmissions to other hospitals. The studies selected for comparison were highly cited papers, but a systematic review was not conducted.

## CONCLUSIONS

Very early surgery for adhesive SBO performed within 12 h of admission did not benefit the patients. A less aggressive approach to the management of frail patients with adhesive SBO would be a wiser option than early surgery prompted by the overestimating of strangulation. There are still insufficient data to draw an overarching conclusion about optimal SBO management. Further randomized controlled trials and studies based on standardized SBO evaluation are needed.

## Data Availability

The datasets generated and/or analyzed during the current study are available from the corresponding author upon reasonable request.
